# Octopamine Shifts the Behavioral Response From Indecision to Approach or Aversion in *Drosophila melanogaster*

**DOI:** 10.3389/fnbeh.2018.00131

**Published:** 2018-07-03

**Authors:** Gerbera Claßen, Henrike Scholz

**Affiliations:** Department of Biology, Institute for Zoology, Biocenter, Albertus-Magnus University of Cologne, Cologne, Germany

**Keywords:** octopamine, Tβh, attraction, aversion, decision making, ethanol attraction, food odor

## Abstract

Animals must make constant decisions whether to respond to external sensory stimuli or not to respond. The activation of positive and/or negative reinforcers might bias the behavioral response towards approach or aversion. To analyze whether the activation of the octopaminergic neurotransmitter system can shift the decision between two identical odor sources, we active in *Drosophila melanogaster* different sets of octopaminergic neurons using optogenetics and analyze the choice of the flies using a binary odor trap assay. We show that the release of octopamine from a set of neurons and not acetylcholine acts as positive reinforcer for one food odor source resulting in attraction. The activation of a subset of these neurons causes the opposite behavior and results in aversion. This aversion is due to octopamine release and not tyramine, since in *Tyramine-β-hydroxylase* mutants (*Tβh*) lacking octopamine, the aversion is suppressed. We show that when given the choice between two different attractive food odor sources the activation of the octopaminergic neurotransmitter system switches the attraction for ethanol-containing food odor to a less attractive food odor. Consistent with the requirement for octopamine in biasing the behavioral outcome, *Tβh* mutants fail to switch their attraction. The execution of attraction does not require octopamine but rather initiation of the behavior or a switch of the behavioral response. The attraction to ethanol also depends on octopamine. Pharmacological increases in octopamine signaling in *Tβh* mutants increase ethanol attraction and blocking octopamine receptor function reduces ethanol attraction. Taken together, octopamine in the central brain orchestrates behavioral outcomes by biasing the decision of the animal towards food odors. This finding might uncover a basic principle of how octopamine gates behavioral outcomes in the brain.

## Introduction

Animals must make constant decisions whether to respond to external sensory stimuli or not to respond. Depending on the internal condition of the animals, they might respond with approach, indifference or aversion. The observed indifference could be due to the inability to perceive the sensory information, the meaninglessness of the information, and/or the inability to decide to which information to respond. Other reasons for showing indifference include the inability of animals to translate the decision into motor output and the inability to execute the locomotion required to fulfill the task. The activation of positive and/or negative reinforcers might bias the behavioral response towards approach or aversion.

In nature, *Drosophila melanogaster* is attracted to ethanol-enriched fermenting food sources (Dudley, 2002; Zhu et al., 2003). For example, female flies search for and prefer fermenting food sources as a substrate to lay their eggs (Richmond and Gerking, 1979; Azanchi et al., 2013). The search for food sources depends on olfactory cues (Chow and Frye, 2009). Given the choice between two similar attractive food odor sources, male and female *Drosophila* are more attracted to ethanol-enriched food odors than to a similar odor without ethanol (Ogueta et al., 2010). The low ethanol concentration that elicits approach functions as a key odorant in the food odor mixture (Giang et al., 2017). The approach depends on the positive reinforcing action of the octopaminergic/tyraminergic neurotransmitter system, as neuronal activation of neurons expressing tyrosine decarboxylase 2 (dTdc2)—the enzyme required for tyramine and indirectly for octopamine synthesis (Cole et al., 2005)—results in attraction (Schneider et al., 2012). The voluntary movement to the site of neuronal activation resembles the voluntary self-administration of electric shocks into the rodent brain that are perceived by the animal as rewarding and uncover the function of reinforcing properties in the brain (Olds and Milner, 1954; Schneider et al., 2012). The optogenetic activating pattern used to stimulate reinforcing tyraminergic/octopaminergic neurons in *Drosophila* brain resemble the high and low frequency of activity observed in the octopaminergic VUMmx1 neuron of the honey bee carrying information of the unconditioned stimulus in olfactory associative appetitive reward learning (Hammer, 1993; Schneider et al., 2012). Activation of the tyraminergic/octopaminergic neurotransmitter system is not only sufficient to cause a bias, but it also might be required for the attraction. *Tyramine-β-hydroxylase* mutants (more specifically the *Tβh^nM18^* mutant)—lacking detectable level of octopamine and with increased TA levels—do not show ethanol-induced site attraction (Monastirioti et al., 1996; Schneider et al., 2012). The loss of attraction is not due to the loss of odorant perception (Schneider et al., 2012), although octopamine might modulate sensory input. For example, octopamine has been shown to reduce the sensory response of bitter-sensing neurons (LeDue et al., 2016).

The octopaminergic neurotransmitter system acts also as a positive reinforcer in other behaviors, such as positive associative olfactory learning and memory in *Drosophila*, (Schwaerzel et al., 2003). However, there is also evidence that the octopaminergic neurotransmitter system might function as a negative reinforcer in the regulation of behavior, as the octopaminergic neurotransmitter system suppresses courtship conditioning (Zhou et al., 2012). Therefore, the octopaminergic neurotransmitter system might do both: function as a positive and negative reinforcer depending on the behavior or condition analyzed. In a more neutral/general way, the octopaminergic neurotransmitter system might function by selecting the right behavioral response or deciding what stimulus to respond to when multiple types of information are presented.

Evidence that octopamine acts as positive and negative regulator is also derived from observations of other insects. In locusts, octopamine release at one site of the ventral nerve cord evokes bouts of rhythmic flight motor activity and at another site suppresses neuronal activity related to oviposition behavior (Sombati and Hoyle, 1984). These results led Sombati and Hoyle (1984) to propose the “orchestration hypothesis” to describe the function of octopamine. For every set of a behavior, a neuronal network exists that can be selectively activated or inhibited by the release of octopamine and therefore allow suppression of opposing behaviors, such as the initiation of flight and suppression of egg laying (Sombati and Hoyle, 1984). However, whether similar selection processes also work in the central brain to modify behavioral output has not been investigated.

To investigate whether the octopaminergic neurotransmitter system is also required in the selection of behavioral programs in the central brain of *Drosophila*, we first addressed whether octopamine can act as negative and positive reinforcer for an odor-guided behavior that involves decision making. To evaluate whether the fly makes a decision, we used a binary choice assay consisting of two odor traps filled with the same food odor to control for the same sensory input. We activated different sets of tyraminergic/octopaminergic neurons using optogenetics and analyzed the consequences of this activation on the choice of the fly to enter a food odor trap. We next addressed whether indeed octopamine and not tyramine mediated these behavioral choices by introducing *Tβh^nM18^* mutants lacking octopamine to the same condition. We show that octopamine functions as a positive and negative reinforcer in odor-evoked behaviors depending on the sets of neurons that are activated. We provide evidence that the activation pattern used for optogenetic activation does not influence the function of the reinforcer. To analyze whether octopamine release shifts the behavioral outcome, we activated the octopaminergic neurons in the presence of a less attractive food odor in comparison to a more attractive ethanol-enriched food odor. We show that the release of octopamine is required to shift the attractiveness of an attractive food odor source to a less attractive food odor source. We further provide evidence that the attraction of ethanol-enriched food odors also requires the release of octopamine by pharmacologically altering octopamine levels and interfering with octopamine receptor function. Taken together, we provide evidence that octopamine is required to select behavioral responses and that the “orchestration of different behavioral circuits underlying behavioral elements” in the central brain is also mediated by octopamine.

## Materials and Methods

### Fly Stocks

Flies were raised and kept on an ethanol-free standard cornmeal/molasses/yeast/agar medium on 12-h/12-h light/dark cycle at 25°C with 60% humidity. Flies carrying transposable elements were backcrossed for at least five generations to the *w^1118^* maintained in the laboratory to isogenize the genetic background. The following fly lines were used: *w^1118^*, *Tβh^nM18^* (Monastirioti et al., 1996), *norpA-; UAS-*ChR2; *UAS-*ChR2 (Schneider et al., 2012), *w^1118^*; *UAS*-ReaChR; *UAS-*ReaChR (Inagaki et al., 2014), *w^1118^; dTdc2-Gal4* (Cole et al., 2005) and *ChAT-Gal80* (Kitamoto, 2002) and *w^1118^*; *6.2_2-Tβh-Gal 4* (Schneider et al., 2012).

### Optogenetic Site Attraction Assay

Flies expressing channelrhodopsin (ChR) or red activatable ChR were raised on food with 200 μl 250 mM all-*trans* retinal (ATR) dissolved in 100% ethanol or 200 μl 100% ethanol in the dark. Fifty three- to five-day-old male flies were collected using CO_2_ anesthesia and recovered in the dark for at least 24 h in the presence of food with either 100 μl 100% ethanol or 100 μl 250 mM ATR. The behavioral experiments were performed as previously described (Schneider et al., 2012). Briefly, flies were given the choice between two odor traps filled with 1.5 ml of apple-mango-juice (Alnatura, Germany GTIN: 4104420071841) for 19 h overnight in the dark at 25°C and 60% humidity.

Both traps were illuminated with two different LEDs with the same intensity ranging from 800 lux to 1700 lux. For the blue light-inducible ChRs, the following two diodes were used: a blue light LED (465–485 nm, Cree, Germany) and a warm white LED (Cree, XLAMP, XR_E LED with 2600–3700K CCT) combined with a blue light filter (high pass filter, 510 nm, HEBO, Aalen, Germany) resulting in yellow light. For the redshifted ChR, the following diodes were used: a blue light LED (465–485 nm, Cree, Germany) and an amber light LED (595 nm, Cree, XRCAMB-L1-0000-00J01). All LEDs repeated the activation pattern of 2 s 40 Hz, 16 s 8 Hz and 2 s 0 Hz for 19 h. The next day, the attraction index (AI) was calculated.

### Olfactory Attraction Assay

The assay was performed as described previously (Ogueta et al., 2010). Briefly, 50 1- to 3-day-old male flies were collected using CO_2_ anesthesia and recovered from the treatment for at least 24 h. The experiments were conducted for 19 h overnight at 25°C and 60% humidity on a cold white light plate or when using ChR on a dark surface. To determine ethanol attraction, flies choose between apple-mango juice and 5% ethanol-enriched apple-mango-juice. Prior to the test, flies were fed with different drugs as follows. The control group was fed on a solution containing 5% sucrose/5% red food color on filter paper. The experimental group received the same food with the drug. If not mentioned otherwise, the flies were starved for 3 h before treatment without any access to water. The concentration of 53 mM octopamine was fed for 1 h (300 μl), 50 mM clonidine for 3 h (200 μl), 200 nM naphazoline for 3 h (200 μl) and 25 mM yohimbine for 2 h (300 μl). Non-starved flies were fed with 3 mM epinastine for 2 days (300 μl, daily remoistening of the filter paper) or with 345 mM tyramine (without 5% sucrose) for 18 h. For the ethanol pre-feeding experiments, flies were fed with 10% ethanol for 30 min (200 μl) after they were starved overnight in the presence of moistened filter paper. Flies were recovered in the presence of moistened filter paper (200 μl) to avoid intoxication during the behavioral experiments for 3.5 h.

### Statistical Analysis

Errors represent the standard error of the mean (SEM). Bars labeled with the letter “a” indicate differences from random choice as determined with a one-sample sign test. Because the data were normally distributed for comparison of two experimental groups, Student’s *T*-test was used. For more than two groups, ANOVA and the Tukey-Kramer *post hoc* test were used. The significance levels are indicated as follows: *P < 0.05, **P < 0.01 and ***P < 0.001.

## Results

**Figure 1:**
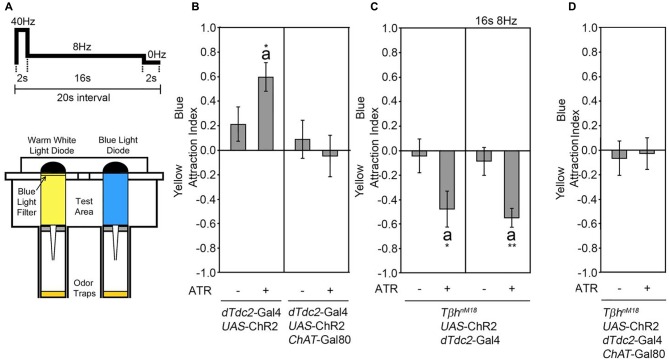
Activation of tyraminergic/octopaminergic/cholinergic neurons results in attraction. **(A)** Schematic of the site attraction assay (modified after Schneider et al., 2012). Two LEDs with the same light intensity but different wavelengths illuminate two similar odor traps with the same flicker frequency of 2 s at 40 Hz, 16 s at 8 Hz and 2 s at 0 Hz. If not otherwise indicated, this pattern was used for activation. The attraction index (AI) describes the fly number in the blue-illuminated odor trap minus the fly number in the yellow-illuminated odor trap in comparison to the total number of flies. **(B)** Neuronal activation using the *UAS-ChR2* under the control of the decarboxylase 2 *(dTdc2)-*Gal4 driver elicits site attraction for the blue illuminated odor trap (AIs of the control and experimental group: 0.21 ± 0.14 and 0.6 ± 0.12, respectively; *n* = 34, 31). Removing the activation of cholinergic neurons by combining the *dTdc2-*Gal4 driver with the *ChAT*-Gal80 driver eliminates site attraction (AIs for the control and experimental group: 0.08 ± 0.16 and −0.05 ± 0.17, respectively; *n* = 25, 23). **(C)** Activation of the *UAS*-ChR2 transgene in a *dTdc2-Gal4*-dependent manner in *Tβh^nM18^* mutants elicits site aversion (AIs of the control and experimental group: −0.05 ± 0.14 and −0.48 ± 0.15, respectively; *n* = 15, 12, and AIs at 8 Hz in the control and experimental group: −0.09 ± 0.11 and −0.55 ± 0.08, respectively; *n* = 20, 19). **(D)** Removal of *UAS*-ChR2 transgene activation by expression of *ChAT*-Gal80 eliminates aversion (AIs of the control and experimental group: −0.07 ± 0.14 and −0.03 ± 0.13, respectively; *n* = 16, 16). Errors are SEM. The letter “a” indicates differences from random choice as determined by the one-sample sign test. Student’s *T*-test was used to determine differences between two groups. *P < 0.05 and **P < 0.05. For data, see Supplementary Table S1.

In a binary choice assay, flies choose between two equal food odor traps (Ogueta et al., 2010). The presence of an attractive key odorant within the food odor biases the decision to move towards and enter the food odor trap containing the key odorant (Giang et al., 2017). The attraction for one odor trap in comparison to an equal second food odor trap can also be induced by activation of tyraminergic/octopaminergic neurons using the *UAS*-ChR2 transgene under the control of the *dTdc2-Gal4* driver (Schneider et al., 2012; Figure 1A). For these experiments, one odor trap is illuminated with blue light, resulting in activation of neurons expressing ChR2 in the presence of ATR, and the second odor trap is illuminated with yellow light with the same intensity to avoid a bias based on difference in light intensity (Figure 1A). To elicit the reinforcing properties of the tyraminergic/octopaminergic neurons, we used a sequence of light stimulation of 40 Hz for 2 s, 8 Hz for 16 s followed by 2 s of no light. This activation pattern mimics the response of the reinforcing VUMmx1 neuron in honey bees (Hammer, 1993; Schneider et al., 2012).

### The Activation of Octopaminergic Neurons Is Sufficient and Required for Attraction

The *dTdc2-*Gal4 line drives transgene expression in at least 137 neurons (Busch et al., 2009). To narrow down the subset of neurons sufficient to induce site attraction, we restricted the expression of the *dTdc2*-Gal4 driver using the *ChAT*-Gal80 driver (Kitamoto, 2002; Figure 1B). The expression of the Gal80 repressor resulted in the expression of transgenes in 34 neurons in the brain, including 3–5 VUM neurons per segment and neurons within the G0b, G2b G3a/AL2, G3b, G5a and G5b, 5c clusters (see Supplementary Figure S1 in Schneider et al., 2012). Activation of these restricted sets of neurons using the *UAS*-ChR2 transgene under the control of the* dTdc2-Gal4* driver combined with the *ChAT*-Gal80 driver did not result in site attraction (Figure 1B). The dTdc2 enzyme is required for the synthesis of tyramine and therefore indirectly required for octopamine synthesis (Cole et al., 2005). The *dTdc2*-Gal4 line drives transgene expression in octopamine-expressing and 41 Tβh-positive neurons in the brain (Busch et al., 2009; Schneider et al., 2012). To address whether octopamine elicits attraction, we performed a similar experiment in *Tβh^nM18^* mutants. The *Tβh^nM18^* mutants lack the Tβh isoform that converts tyramine into octopamine and have no detectable levels of octopamine, but 8-fold significantly increased levels of tyramine (Monastirioti et al., 1996). Activation of *dTdc2-*Gal4-targeted neurons in a *Tβh^nM18^* mutant background lacking octopamine resulted in loss of attraction and significant site aversion using two different activation frequencies (Figure 1C). This result suggests that octopamine release mediated attraction. The observed aversion might be due to increased tyramine levels or other neurotransmitter that were co-expressed in tyraminergic/octopaminergic neurons. To test whether activation of acetylcholine transferase (ChAT)-positive neurons elicits aversion, expression of the *dTdc2*-Gal4 driver in the *Tβh^nM18^* mutant background was restricted using the *ChAT*-Gal80 driver (Figure 1D) to reduce transgene expression to 16 neurons in the brain, including a subset of VUM neurons and neurons of the G3a/AL2 and G3b cluster (Schneider et al., 2012; see Supplementary Figure S1). Repression of activation in cholinergic neurons resulted in a loss of aversion, indicating that activation of ChAT-positive neurons mediated the aversion. Taken together, the results suggest that octopamine release from acetylcholine co-expressing neurons mediates attraction.

### Activation of a Subset of Octopaminergic Neurons Is Sufficient and Required for Aversion

**Figure 2:**
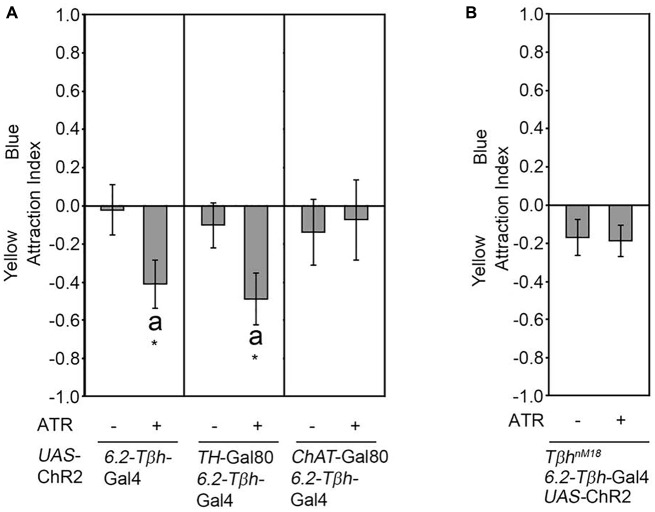
Octopamine release causes aversion. **(A)** Neuronal activation under the control of the *6.2-Tβh-*Gal4 driver elicits site aversion (AIs of the control and experimental group: 0.02 ± 0.14 and −0.41 ± 0.13, respectively; *n* = 22, 19). Removal of the activation of dopaminergic neurons by combining the *6.2-Tβh-*Gal4 driver with the *TH*-Gal80 driver does not influence aversion (AIs for the control and experimental group: 0.1 ± 0.12 and −0.49 ± 0.14, respectively; *n* = 14, 14). Removing ChAT positive neurons by using the *ChAT*-Gal80 in combination with the *6.2-Tβh-*Gal4 driver resulted in loss of aversion (AIs of control and experimental group: −0.14 ± 0.17 and −0.08 ± 0.21 respectively; *n* = 13, 11). **(B)** Activation of the *UAS*-ChR2 transgene under the control of 6.2.-*Tβh-Gal4* in *Tβh^nM18^* mutants eliminates aversion (AIs of the control and experimental group: −0.17 ± 0.1 and −0.19 ± 0.08, respectively; *n* = 20, 19). For activation, the flicker frequency of 2 s at 40 Hz, 16 s at 8 Hz and 2 s at 0 Hz was used. Errors are SEM. Differences from random choice were determined using the one-sample sign test and are indicated by the letter “a”. Student’s *T*-test was used to determine differences between two groups. *P < 0.05. For data, see Supplementary Table S2.

We next addressed whether activation of a subset of neurons included in the expression pattern of the *dTdc2*-Gal4 driver line also resulted in attraction. Therefore, we expressed the ChR2 transgene in three VUMa4 neurons in the SOG using the *6.2-Tβh-*Gal4 driver (Figure 2). The *6.2-Tβh-*Gal4 driver targets transgene expression in the three VUMa4 neurons and also to non-Tβh positive neurons (Schneider et al., 2012). In contrast to the whole set of VUM neurons included in the expression pattern of the *dTdc2-*Gal4 driver, the activation of neurons in *6.2-Tβh-*Gal4 dependent manner resulted in significant aversion to the site of blue light activation (Figure 2A). Activation of a subset of dopaminergic neurons using the *TH*-Gal4 driver resulted in aversion (Schneider et al., 2012). To exclude that the observed aversion was due to activation of dopaminergic neurons, we combined the *6.2-Tβh-*Gal4 with the *TH*-Gal80 driver to suppress expression in putative dopaminergic neurons (Figure 2A). Here, neuronal activation still resulted in significant aversion (Figure 2A). Restriction of the Gal4 expression pattern of the *6.2-Tβh-*Gal4 driver to non-cholinergic neurons using the *ChAT*-Gal80 driver resulted in loss of aversion showing that activation of ChAT positive neurons mediate aversion (Figure 2A). To investigate whether the observed aversion was also regulated by octopamine release, we activated neurons targeted by the *6.2-Tβh-GAL4* driver in *Tβh^nM18^* mutants lacking octopamine (Figure 2B). Here, the activation did not elicit site aversion or attraction. Therefore, the results suggests that octopamine can also mediate aversion depending on the sets of octopaminergic neurons activated.

### The Frequency and Intensity of Light Activation Influences the Behavioral Outcome

**Figure 3:**
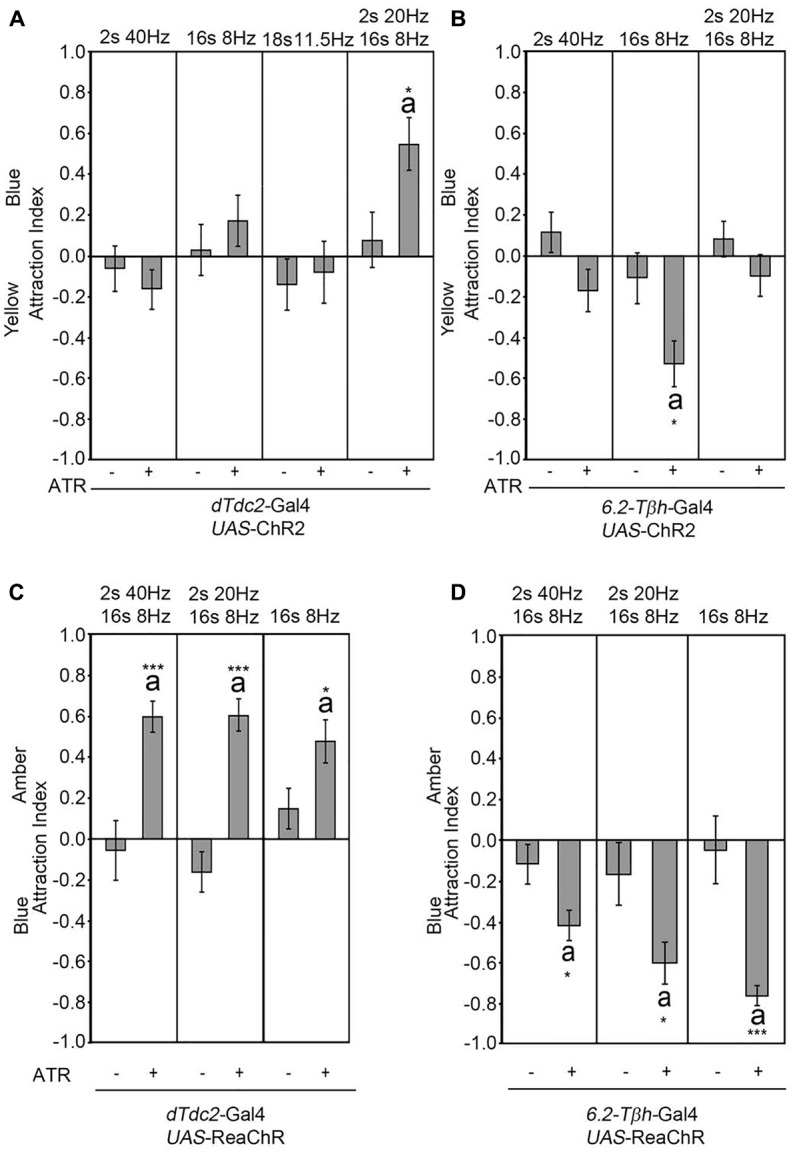
Frequency-dependent activation results in attraction. Different flicker patterns indicated above the panel were used to activate neurons in a *dTdc2-*Gal4-dependent manner **(A,C)** and *6.2-Tβh-*Gal4-dependent manner **(B,D)**. Only activation with a flicker of 2 s at 20 Hz, 16 s at 8 Hz followed by 2 s of silence continues to elicit site attraction (A, AIs of the control and experimental group: −0.08 ± 0.14 and 0.55 ± 0.13, respectively; *n* = 27, 23). **(B)** The 8 Hz pattern is sufficient to elicit site aversion when used to activate neurons (AIs of the control and experimental group: −0.11 ± 0.12 and −0.53 ± 0.11, respectively; *n* = 20, 14). **(C)** Activation with *UAS-ReaChR* in a *dTdc2-*Gal4 dependent manner leads to site attraction (2 s at 40 Hz, 16 s at 8 Hz activation pattern: AIs of the control and experimental group: −0.06 ± 0.15 and 0.6 ± 0.08, respectively; *n* = 18, 18; 2 s at 20 Hz, 16 s at 8 Hz activation pattern: AIs of the control and experimental group: −0.17 ± 0.1 and 0.6 ± 0.08, respectively; *n* = 13, 7; 16 s at 8 Hz activation pattern: AIs of the control and experimental group: 0.15 ± 0.1 and 0.47 ± 0.11, respectively; *n* = 12, 8). **(D)** Activation with *UAS-ReaChR* in a *6.2-Tβh-*Gal4-dependent manner results in site aversion (2 s at 40 Hz, 16 s at 8 Hz activation pattern: AIs of the control and experimental group: −0.12 ± 0.1 and −0.42 ± 0.08, respectively; *n* = 23, 20; 2 s at 20 Hz, 16 s at 8 Hz activation pattern: AIs of the control and experimental group: −0.17 ± 0.2 and −0.61 ± 0.1, respectively; *n* = 23, 16; 16 s at 8 Hz activation pattern: AIs of the control and experimental group: −0.05 ± 0.17 and −0.77 ± 0.05, respectively; *n* = 18, 17). Errors are SEM. Differences from random choice as determined by the one-sample sign test are labeled with the letter “a”. Student’s *T*-test was used to determine differences between two groups with significance levels as follows: *P < 0.05, ***P < 0.001. For data, see Supplementary Table S3.

The frequency used to activate the *UAS*-ChR2 transgene mimicked the frequency used to depolarize the VUMmx1 neuron in honey bees (Hammer, 1993; Schneider et al., 2012). The depolarization with electrodes in honey bees and the activation of the UAS-ChR2 transgene in *Drosophila* using optogenetics with this frequency was sufficient to elicit the reinforcer (Hammer, 1993; Schneider et al., 2012). To further investigate whether the observed site attraction or site aversion was caused by a specific activation frequency, we used different light flicker frequencies with a constant intensity to activate the *UAS*-ChR2 transgene in a *dTdc2-GAL4*-dependent manner and analyzed the consequence of this activation on site attraction (Figure 3). Neither the short pulse of 40 Hz nor the long pulse of 8 Hz was sufficient to result in site attraction (Figure 3A). We next tested the average frequency of 11.5 Hz. The flicker pattern still did not result in site attraction. A reduction of 40–20 Hz within the 40–8 Hz pattern was sufficient to elicit a significant attraction. The observed frequency-dependent site attraction suggested that either a specific frequency or the kinetics of the transgene influenced the behavioral outcome. We next tested whether there was a similar frequency dependence behavioral outcome when using the *6.2-Tβh*-Gal4 driver (Figure 3B). Activation of the *UAS*-ChR2 transgene with an 8-Hz pulse followed by 4 s in the dark in a *6.2-Tβh*-Gal4-dependent manner was sufficient to elicit site aversion. However, neither the reduction to 20 Hz for 2 s followed by 8 Hz for 16 s nor the activation by only 40 Hz resulted in site aversion. Next, we addressed whether the intensity of the light influenced the activation of the transgene (Supplementary Figure S1, Table S6). The flicker sequence used for activation was kept constant with a pattern of 40 Hz for 2 s followed by 8 Hz for 16 s. Changing the light intensity for neuron activation resulted in site attraction in a *dTdc2-GAL4*-dependent manner; however, the degree of attraction as measured in AI varied from 0.3 to 0.6. Changing the light intensity for neuron activation in a *6.2-Tβh*-Gal4-dependent manner only resulted in differences between the control and experimental group when neurons were activated with 1200 lux (Supplementary Figure S1B). Thus, the activation frequencies of the *UAS*-ChR2 transgene and light intensities influence the behavioral outcome.

To address, independent of the kinetics of the blue light sensitivity of the ChR2 transgene, whether neuronal activation of tyraminergic/octopaminergic neurons induced site attraction, we used the *UAS*-ReaChR transgene for neuronal activation (Figure 3C; Inagaki et al., 2014). The ReaChR encodes a redshifted channel rhodopsin variant with an activation light spectrum between orange and red, higher photocurrents and faster kinetics (Lin et al., 2013). Activation of neurons using the *UAS-*ReaChR transgene in a *dTdc2-*Gal4-dependent manner was sufficient to elicit site attraction, when the tyramine/octopamine-positive neurons were activated by amber light (Figure 3C). All tested activation patterns resulted in attraction. In addition, activation of neurons with three different flicker frequencies in a *6.2-Tβh*-Gal4-dependent manner caused site aversion (Figure 3D). Therefore, the activation pattern of the neurons did not appear to be frequency-dependent but rather based on the kinetic of the transgene. Independent of the transgene, the activation of neurons in a *dTdc2-*Gal4-dependent manner was sufficient to elicit site attraction, and activation of neurons in a *6.2-Tβh*-Gal4-dependent manner was sufficient to elicit site aversion.

### Octopaminergic Neurons Are Required to Switch the Behavioral Response

**Figure 4:**
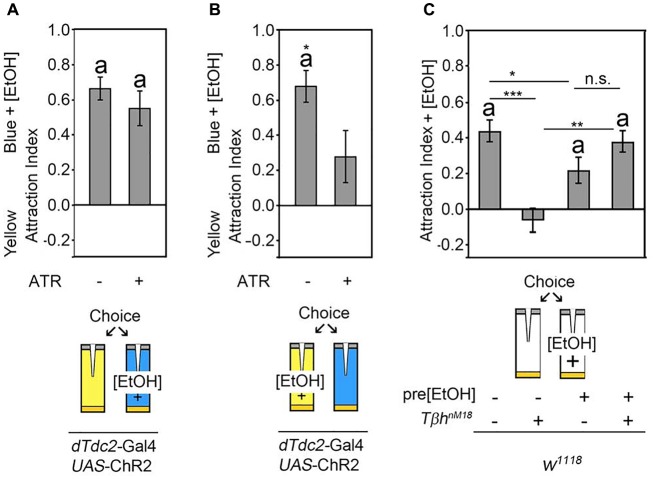
Octopaminergic neurotransmitter shifts behavioral outcomes. **(A)** Neuronal activation using the blue light-sensitive *UAS*-ChR2 transgene in a *dTdc2-Gal4-*dependent manner results in attraction to the blue light-illuminated odor trap. Addition of 5% ethanol to the blue light-illuminated trap did not change this attraction significantly (AIs of the control and experimental group: 0.66 ± 0.07 and 0.55 ± 0.1, respectively, *n* = 25, 17). **(B)** Addition of 5% ethanol to the yellow light-illuminated trap results in a significant reduction of the attraction to the blue light-illuminated food odor trap (AIs of the control and experimental group: 0.68 ± 0.09 and 0.28 ± 0.15, respectively, *n* = 20, 18). **(C)** Flies normally prefer the 5% ethanol-enriched food odor trap over the plain food odor trap, and *Tβh^nM18^* mutants differ significantly from the control in their behavior and do not show attraction. Pre-feeding control flies with 10% ethanol-enriched sucrose solution results in a similar degree of attraction to the 5% ethanol-containing food odor trap. Ethanol pre-feeding in *Tβh^nM18^* mutants results in a significant attraction to the 5% ethanol-containing food odor trap (AIs for *w^*1118*^*: 0.44 ± 0.06 and with EtOH: 0.22 ± 0.08; for *w^*1118*^, Tβh^*nM18*^*: −0.07 ± 0.07 and with EtOH: 0.38 ± 0.06; *n* = 33, 31, 17, 20). The errors are SEM. The differences from random choice were determined using the one sample- sign test, and significance is indicated by the letter “a”. Student’s *T*-test was applied to determine differences between two groups, and ANOVA followed by Tukey *post hoc* analyses were used for comparisons among more than two groups. Significant differences are indicated as follows: *P < 0.05, **P < 0.01 and ***P < 0.001. For data, see Supplementary Table S4.

The release of octopamine from acetylcholine co-expressing neurons mediates attraction for the site of neuronal activation. The site attraction could be due to reinforcement or due to a behavioral switch. To test whether the octopamine release reinforced the positive association with one site of the behavioral choice paradigm, we wanted to analyze whether octopamine release could increase a pre-existing attraction. Normally flies show attraction to 5% ethanol-enriched food odors when given the choice between food odor and ethanol-enriched food odor (Schneider et al., 2012). To analyze whether octopamine release increased this attraction, flies expressing the *UAS*-ChR2 transgene under the control of the *dTdc2*-Gal4 driver were offered a choice between a food odor trap and a 5% ethanol-enriched food odor trap that was illuminated with neuron-activating flickering blue light (Figure 4A). Activation of tyraminergic/octopaminergic neurons did not significantly increase the attraction for the ethanol-enriched food odor trap. To test whether the activation of tyraminergic/octopaminergic neurons would switch a preexisting ethanol attraction to the site of blue light activation, we enriched the yellow light-illuminated food odor trap with ethanol (Figure 4B). Here, as expected, control flies were significantly more attracted to the ethanol-enriched yellow-illuminated food odor trap. The activation of tyraminergic/octopaminergic neurons significantly suppressed this ethanol attraction, indicating that tyraminergic/octopaminergic neurons are not directly involved in attraction rather mediating the switch between two choices.

To independently address whether octopamine release mediated switches between behavioral elements rather than being directly involved in the execution of behavior, we reanalyzed the choice behavior of *Tβh^nM18^* mutants (Figure 4C). In contrast to control flies, *Tβh^nM18^* mutants failed to show attraction to 5% ethanol-enriched food odors (Schneider et al., 2012; Figure 4C). To address whether *Tβh^nM18^* were unable to show attraction *per se*, we pre-exposed flies to ethanol by feeding a 10% ethanol-enriched 5% sucrose solution for 30 min. After 3.5 h of recovery, the attraction to ethanol-enriched food odors was analyzed in the binary choice assay (Figure 4C). Ethanol pre-fed control flies showed an attraction to ethanol-enriched food odors, and ethanol pre-feeding of *Tβh^nM18^*mutants resulted in a similar significant ethanol attraction. These results showed that ethanol pre-feeding to *Tβh^nM18^* mutants restored the attraction to ethanol-containing food odors. Thus, *Tβh^nM18^* mutants were able to develop the attraction but failed to choose ethanol-enriched food odors in an ethanol naïve choice situation over non-ethanol-containing food odors. Taken together, activation of the octopaminergic/tyraminergic neurotransmitter system can shift the attraction, and *Tβh^nM18^* fail to show ethanol attraction because they cannot shift their behavioral response.

### Octopamine Is Required for Ethanol Attraction

**Figure 5:**
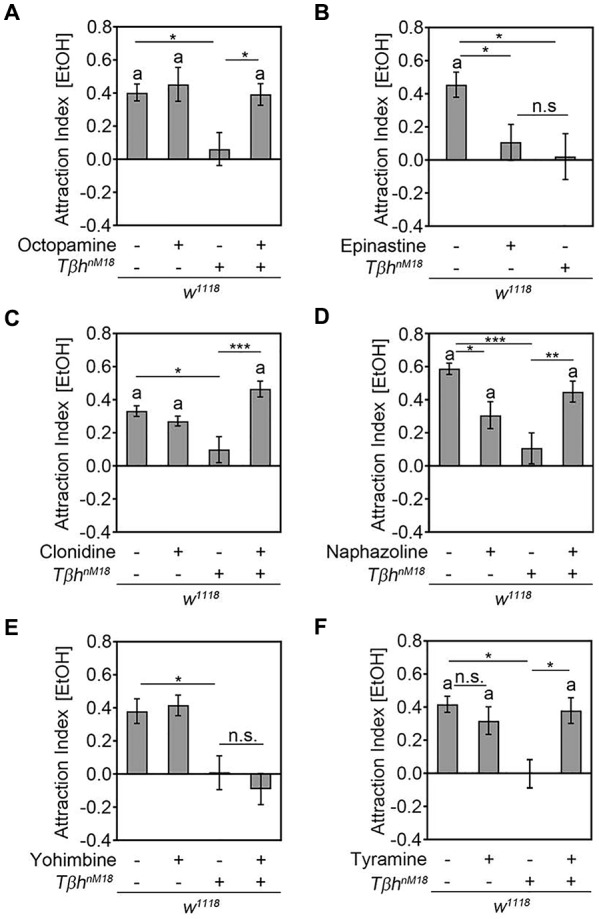
Octopamine is required for ethanol attraction. **(A)** Feeding with 53 mM octopamine restores the loss of attraction to ethanol-containing food odors in *Tβh^nM18^* mutants (AIs for *w^*1118*^*: 0.4 ± 0.05 and with octopamine: 0.45 ± 0.11; for *w^*1118*^, Tβh^*nM18*^*: 0.06 ± 0.1 and with octopamine: 0.39 ± 0.07; *n* = 25, 21, 19, 22). **(B)** Blocking OA receptors by feeding 3 mM epinastine eliminates the attraction of control flies similarly to the observed loss of attraction in *Tβh^nM18^* mutants (AIs for *w^*1118*^*: 0.45 ± 0.08 and with epinastine: 0.11 ± 0.11; for *w^*1118*^, Tβh^*nM18*^*: 0.02 ± 0.15; *n* = 29, 28, 19). **(C)** *Tβh^nM18^* mutants fed with 50 mM clonidine show a significant attraction to ethanol-containing food odors (AIs for *w^*1118*^*: 0.33 ± 0.04 and with clonidine: 0.27 ± 0.03; for *w^*1118*^, Tβh^*nM18*^*: 0.1 ± 0.08 and with clonidine: 0.47 ± 0.05; *n* = 30, 28, 29, 31). **(D)** Feeding 200 nM naphazoline to *Tβh^nM18^* mutants restores the loss of attraction to ethanol-containing food odors and significantly reduces the attraction of the control group (AIs for *w^*1118*^*: 0.59 ± 0.04 and with naphazoline: 0.31 ± 0.08; for *w^*1118*^, Tβh^*nM18*^*: 0.11 ± 0.09 and with naphazoline: 0.45 ± 0.07; *n* = 32, 33, 29, 33). **(E)** Blocking TA receptors by feeding with 25 mM yohimbine does not alter the attraction to ethanol-containing food odors (AIs for *w^*1118*^*: 0.38 ± 0.08 and with yohimbine: 0.41 ± 0.07; for *w^*1118*^, Tbh^*nM18*^*: 0.01 ± 0.11 and with yohimbine: 0.09 ± 0.1; *n* = 40, 40, 21, 24). **(F)** Increasing tyramine levels in control flies do not significantly alter attraction, but they significantly induce attraction in *Tβh^nM18^* mutants (AIs for *w^*1118*^*: 0.42 ± 0.05 and with tyramine: 0.32 ± 0.09; for *Tβh^nM18^*: −0.01 ± 0.09 and with tyramine: 0.38 ± 0.08). Errors are SEM, and the letter “a” indicates differences from random choice as determined by the one-sample sign test. Student’s *T*-test was used to determine differences between two groups and ANOVA followed by the Tukey *post hoc* test for more than two groups. *P < 0.05, **P < 0.01, ***P < 0.001. For data, see Supplementary Table S5.

To determine whether the loss of ethanol attraction in ethanol naïve *Tβh^nM18^* was due to the loss of octopamine, we performed pharmacological experiments (Figure 5). First, we fed *Tβh^nM18^* mutants with 53 mM octopamine—a concentration that restored the egg laying defect of the *Tβh^nM18^* mutants (Monastirioti et al., 1996)—and analyzed their ethanol attraction (Figure 5A). In addition, we fed control flies with octopamine to investigate whether increased octopamine levels could increase attraction. Feeding with OA restored the loss of ethanol attraction in *Tβh^nM18^* mutants to control levels, but it did not alter the attraction of *w^*1118*^* flies (Figure 5A). To independently confirm that octopamine signaling was required for attraction, *w^*1118*^* flies were fed with 3 mM of the antagonist epinastine for octopamine receptors, a concentration that has been shown to effectively interrupt TfAP-2-induced hyperactivity in *Drosophila* (Williams et al., 2014). Epinastine-fed control flies showed a similar significant loss of ethanol attraction to *Tβh^nM18^* mutants (Figure 5B). To confirm that octopamine receptor signaling was required for ethanol attraction, we fed β*h*^*nM18*^ mutant and control flies with two octopamine receptor agonists—50 mM clonidine or alternatively 200 nM naphazoline (Evans, 1981; Evans and Maqueira, 2005)—and investigated the effects on attraction (Figures 5C,D). The octopamine receptor agonist naphazoline and clonidine should restore octopamine receptor signaling in *Tβh^nM18^* mutants. Clonidine turned the loss of ethanol attraction of *Tβh^nM18^* mutants into attraction, but it did not influence the attraction of control flies (Figure 5C). Naphazoline also turned the loss of attraction into ethanol attraction in* Tβh^*nM18*^* mutants (Figure 5D). However, naphazoline also altered the attraction in control animals by reducing the attraction, suggesting that too much activation of octopamine receptors might result in aversion or that naphazoline activates additional receptors that mediate aversion. To address the putative function of increased tyramine levels on ethanol attraction, tyramine receptor signaling was blocked in control flies and *Tβh^nM18^* mutants by feeding with the tyramine receptor antagonist yohimbine. Feeding with 25 mM yohimbine—a concentration that rescues the *Tβh^nM18^* mutant phenotype in flight initiation and maintenance (Brembs et al., 2007)—did not alter the attraction to ethanol in control or in mutant flies (Figure 5E). To independently address whether increased tyramine levels altered ethanol attraction, 345 mM tyramine was fed to control flies, and the effects on attraction to ethanol were analyzed (Figure 5F). Control flies fed with tyramine did not show a significantly reduced attraction to ethanol-containing food odors. Feeding tyramine to the* Tβh^*nM18*^* mutant significantly induced attraction to ethanol-containing food odors, suggesting that feeding with tyramine was effective and might function at high levels as an agonist for octopamine receptors, or alternatively, activation of tyramine receptors might also promote the induction of attraction to ethanol-containing food odors. Taken together, octopamine is required for the attraction to ethanol, and increased TA levels in *Tβh^nM18^* mutants are not responsible for the loss of attraction.

## Discussion

**Figure 6:**
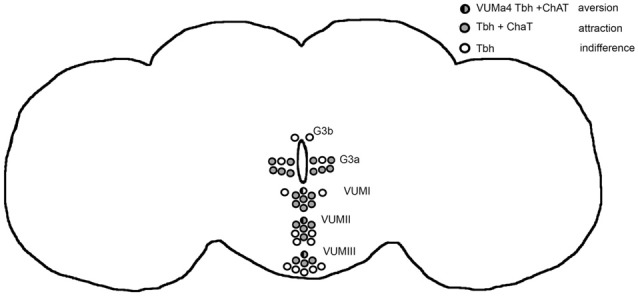
Model for octopamine induced attraction and aversion. The circles highlight the Tbh positive neurons targeted by the *dTdc2-*Gal4 driver in the adult brain (Schneider et al., 2012). The Tbh is the rate limiting enzyme for octopamine synthesis (Monastirioti et al., 1996). Activation of these neurons using *UAS*-ChR2 elicits site attraction (Figure 1). When the Gal4 expression of the driver is restricted by co-expression of *ChAT*-GAL80 driver the flies choose both odor traps equally (Figure 1). The neurons that are not affected by the ChAT-Gal80 repressor are indicated in open circles. They only express Tbh and activation of these neurons results in indifferences (Figure 1). The ChAT and Tbh expressing neurons are indicated by gray circles and they are responsible—when activated—to mediate attraction. Within these set of positive neurons, the activation of the VUMa4 neuron (indicated by a circle that is half black and half gray) results in aversion (Figure 2). The VUMa4 neurons are Tbh positive (Schneider et al., 2012). Since the ChAT-Gal80 repressor eliminates the aversion caused by activation of the 6.2 *Tbh*-Gal4 driver, the VUMa4 neuron is also ChAT positive (Figure 2). The activation of the VUMa4 neuron results in aversion, but when the VUMa4 neuron is activated together with other octopaminergic neurons, attraction occurs. This result is consistent with the idea that the activation of a second set of octopaminergic neurons can overrule the octopamine-induced aversion by the VUMa4 neuron. For data, see Supplementary Table S6.

The release of octopamine from acetylcholine co-expressing tyraminergic/octopaminergic neurons is necessary to induce a bias in a behavioral choice between two identical food odor traps, resulting in attraction for one odor trap. The release of tyramine or acetylcholine from the same set of cholinergic neurons results in aversion. In addition, the release of octopamine from VUMa4 neurons acts as a negative reinforcer when flies choose between two identical food odor traps and causes aversion. Therefore, octopamine mediates aversion and attraction depending on the set of neurons activated. In Figure 6 we provide an overview summarizing these results. The behavioral outcome of neuronal activation depends on the kinetics of the channelrhodopsin transgene used for activation. Nevertheless, independent of the transgene, the activation of tyraminergic/octopaminergic neurons is sufficient to elicit site attraction, and the activation of VUMa4 neurons is sufficient to elicit site aversion. Given the choice between an attractive ethanol-enriched food odor and a less attractive food odor, the activation of tyraminergic/octopaminergic neurons is able to shift the attraction to the less attractive food odor. The induction of attraction for ethanol-enriched food odors in a binary choice also requires the function of octopamine, supporting the requirement for octopamine to trigger the behavior towards approach, e.g., ethanol attraction.

### Octopamine Acts as Positive and Negative Reinforcer

In *Drosophila*, octopamine has been shown to function as a positive reinforcer in several behaviors that involve the processing, evaluation and behavioral response of olfactory information. Innate attraction to ethanol-containing food odors requires the activation of tyraminergic/octopaminergic neurons, and mutants lacking octopamine fail to show attraction (Schneider et al., 2012). In more complex behaviors such as olfactory learning and memory, octopamine functions as a positive reinforcer, since *Tβh^nM18^* mutants fail to show a positive association or attraction to a previous sucrose rewarded odor (Schwaerzel et al., 2003). Similar to the adult nervous system, in the developing nervous system of 3^rd^ instar larvae, activation of tyraminergic/octopaminergic neurons substitutes for reward in olfactory larval learning, and the activity of tryaminergic/octopaminergic neurons is required for olfactory appetitive learning and memory (Schroll et al., 2006; Selcho et al., 2014). Here, we show that activation of tyraminergic/octopaminergic neurons is sufficient to bias the decision toward one of two similar odor traps and that *Tβh^nM18^* mutants fail to show attraction upon activation of the same set of neurons (Figure 1). Octopamine release from VUMa4 neurons results in aversion, indicating that octopamine functions as negative reinforcer in an olfactory choice situation (Figure 2). Eliminating octopamine release from 6.2-*Tβh*-Gal4-targeted neurons by introducing *Tβh^nM18^* mutants clearly eliminated aversion, showing that octopamine release is required for aversion. Another observation supporting that octopamine acts as a negative reinforcer was derived from the observation that the octopamine receptor agonist naphazoline reduced attraction in control flies (Figure 5D). There is evidence that the octopaminergic system might also function as negative reinforcer in other behaviors. In olfactory aversive learning and memory, when an odor is paired with an electric shock, *Tβh^nM18^* mutants show reduced learning abilities, indicating an impairment of the negative reinforcing function of the electric shock (Iliadi et al., 2017). Furthermore, activation of tyraminergic/octopaminergic neurons suppresses courtship conditioning, another form of aversive associative learning (Zhou et al., 2012).

On the cellular level, opposing neuronal functions for octopamine have been previously observed. For example, in the ventral nerve cord of locusts, octopamine release causes, at one site, bouts of rhythmic flight motor activity and, at another site, suppression of neuronal activity related to egg laying (Sombati and Hoyle, 1984). The opposing roles of octopamine might also be explained by the activation of different classes of octopamine receptors that either activate or reduce neuronal activity, both of which have been identified in *Drosophila melanogaster* (Evans and Maqueira, 2005). In addition to the negative reinforcing function of octopamine, an additional neurotransmitter mediates aversion in the *dTdc2*-Gal4-targeted neurons, since the introduction of *Tβh^nM18^* mutants resulted in significant aversion. One possible candidate for this is tyramine. For example, feeding the tyramine receptor antagonist yohimbine to *Tβh^nM18^* mutant larvae partially restored the observed reduced locomotion in the mutants, suggesting that the reduced locomotion was due to an increased tyramine level and that tyramine might counteract the function of octopamine (Saraswati et al., 2004). However, feeding with the tyramine receptor antagonist yohimbine did not interfere with attraction to ethanol in control flies, nor did it alter the loss of attraction in *Tβh^nM18^* mutants, suggesting that tyramine is not responsible for the observed aversion. Furthermore, feeding tyramine to control flies did not suppress the attraction to ethanol-enriched food odors (Figure 5F). Rather, feeding tyramine to *Tβh^nM18^* mutants resulted in attraction, suggesting that high levels of tyramine might also function also as a positive reinforcer. Another candidate is acetylcholine. Neuronal activation of neurons in a *dTdc2*-Gal4-dependent manner might also co-release other neurotransmitters that negatively regulate attraction. Not all neurons targeted by the *dTdc2*-Gal4 line are octopaminergic or Tβh-positive (Busch et al., 2009; Schneider et al., 2012), and *ChAT*-Gal80 represses *dTdc2*-Gal4-dependent transgene expression (Schneider et al., 2012). Consistent with this observation, some of the targeted VUM neurons express ChAT (Sayin et al., 2018). However, activation of VUMa4 neurons in *Tβh^nM18^* mutants eliminates the aversion, clearly indicating that octopamine, in addition to possible other neurotransmitter systems, might induce aversion. Taken together, octopamine functions as a positive and negative reinforcer in response to olfactory cues such as food odors.

### Octopamine Biases Behavioral Outcomes

How does a neurotransmitter mediate both negative and positive reinforcement to an olfactory cue? In associative olfactory short-term learning and memory, dopamine mediates two different sets of neurons: positive and negative reinforcing properties (Claridge-Chang et al., 2009; Aso et al., 2010; Liu et al., 2012). The same phenomenon might also be true for octopaminergic neurons. Octopamine-dependent activation of the VUMa4 neuron results in aversion, but when the VUMa4 neuron is activated together with other octopaminergic neurons, attraction occurs. This result indicates that the activation of a second set of octopaminergic neurons can overrule the octopamine-induced aversion by the VUMa4 neuron. Such an attraction might be the primary behavioral outcome when octopamine release is depleted, as supported by the observation that *Tβh* mutants with no detectable levels of octopamine fail to show attraction and feeding control flies with the octopamine receptor antagonist epinastine results in a reduction of ethanol-induced attraction rather than increased attraction. Feeding the agonist of octopamine—naphazoline- to control flies also reduces the attraction to ethanol enriched food odor (Figure 5D). This could be due to the activation of octopamine dependent negative reinforcement resulting in aversion.

If octopamine mediates both attraction and aversion to olfactory cues, then a situation must arise where octopamine-deficient flies respond with indecision in a choice situation, which is indeed the case. *Tβh^nM18^* mutants fail to show attraction to ethanol-enriched food sources. The indecision to choose between the two odor sources might depend on the inability to detect ethanol within food odors, the lack of a positive reinforcer, inability to make a decision or failure to perform the motor skills required for the task. *Tβh^nM18^* mutants sense ethanol and food odors and can distinguish between them (Schneider et al., 2012), eliminating the possibility that failures in odor perception account for the indifference. During olfactory learning and memory, odor ethanol functions as a positive reinforcer (Kaun et al., 2011), and *Tβh^nM18^* mutants show attraction to ethanol-containing food odors when they are pre-exposed to ethanol (Figure 4C), showing that the mechanism resulting in positive reinforcement is still functional in *Tβh^nM18^* mutants. Furthermore, *Tβh^nM18^* mutants still enter food odor traps, showing that the motor ability of the flies and the motivation to seek food is still intact. Consistent with a function in decision making, the activation of octopaminergic/tyraminergic neurons shifts the attraction from the ethanol-enriched food odor to a less attractive odor (Figure 4B) and therefore biases the behavioral outcome.

Are there other behaviors where flies normally must decide to respond to odor information and where *Tβh^nM18^* mutants fail to make the right decision or correct response? After exposure to a novel olfactory stimulus such as ethanol, normal flies show an increased startle response as measured by an increase in locomotion (Scholz, 2005). The *Tβh^nM18^* mutants not only fail to show a startle response but also show a repression of locomotor activity that can be contributed to the lack of octopamine, mimicking a freezing response. The immobility is turned into activity when the ethanol stimulus is sufficiently strong (Scholz, 2005). Another example that involves a behavioral choice is the associative appetitive and aversive olfactory learning paradigm that is analyzed in the Tully-Quinn paradigm (Tully and Quinn, 1985). In this paradigm, flies must demonstrate what they learned in a binary choice between two odors. Before the experiments, the odor concentrations are balanced so that they evoke a similar behavioral response to ensure that the strength of the presented odor stimuli is comparable. *Tβh^nM18^* mutants fail to respond to the positive reinforced odor with attraction within the given time, but they respond with aversion to the negative reinforced odor (Schwaerzel et al., 2003) or only respond in a reduced manner (Iliadi et al., 2017).

The difference between appetitive and aversive learning defects in *Tβh^nM18^* mutants might be due to the strength of the reinforcer. The lack of comparability of the strength of the reinforcer between aversive and appetitive olfactory learning and memory is also observed in the degree of association formed between the odor and the reinforcer, as reflected in differences in the learning score. Flies show lower learning scores when a positive reinforcer, such as sucrose, is paired with an odor compared with when a negative reinforcer, such as an electric shock, is paired (Schwaerzel et al., 2003). The influence of the threshold of the reinforcing stimulus is also observed in the behavioral response of *Tβh^nM18^* mutants when they are pre-exposed to ethanol and respond with ethanol attraction in the binary choice paradigm (Figure 4C). Here, the pre-exposure to ethanol odor is sufficiently strong to elicit attraction. In summary, octopamine orchestrates the behavioral response to odors. It remains to be investigated whether this finding holds true for other sensory information, such as taste and vision, and how octopamine integrates different external and/or internal information to bias the behavioral outcome.

## Ethics Statement

The animal studies including the model organism *Drosophila melanogaster* were conducted in agreement with the regulations of the DFG and the land North Rhine Westphalia.

## Author Contributions

GC and HS: conception and design of the work; acquisition, data analysis and interpretation. GC: original draft—writing. HS: supervision, project administration and funding acquisition.

## Conflict of Interest Statement

The authors declare that the research was conducted in the absence of any commercial or financial relationships that could be construed as a potential conflict of interest.

## Supplementary Material

FIGURE S1 Effect of light intensity on behavioral outcome. The same flicker frequency of 2 s at 40 Hz, 16 s at 8 Hz was used for all experiments, but the intensity of both LEDs was changed simultaneously. **(A)** Different intensities indicated above the panel were used to activate neurons with a blue light-sensitive *UAS*-ChR2 under the control of the *dTdc2-Gal4* driver. All the used intensities resulted in attraction for the site of activation (AIs for the 1700 lux control and experimental group: −0.07 ± 0.12 and 0.32 ± 0.1, *n* = 20, 20; for 1000 lux: 0.21 ± 0.14 and 0.6 ± 0.12, *n* = 34, 31; for 800 lux: 0.02 ± 0.09 and 0.43 ± 0.1, respectively, *n* = 39, 33). **(B)** Neurons activated with a blue light-sensitive *UAS-ChR2* under the control of the 6.2-*Tβh*-Gal4 driver with different intensities showed significant aversion when illuminated with 1200 lux, but not 1700 lux or 1000 lux (AIs for the 1700 lux control and experimental group: −0.31 ± 0.14 and −0.62 ± 0.11, *n* = 20, 16; for 1200 lux: −0.02 ± 0.13 and −0.41 ± 0.13; *n* = 22, 19 and for 1000 lux: 0.12 ± 0.13 and −0.02 ± 0.12, respectively; *n* = 21, 19). **(C)** Activation with the amber-sensitive *UAS-ReaChR* in a *dTdc2*-Gal4-dependent manner leads to site attraction for the amber-illuminated site (AIs for the 1200 lux AIs of control and experimental group: −0.06 ± 0.15 and 0.6 ± 0.08, *n* = 18, 18; AIs for the 800 lux 0.21 ± 0.11 and 0.1 ± 0.14, respectively, *n* = 14, 5). Errors bars are SEM. The one-sample sign test was used to determine differences from random choice, and significant differences are labeled with the letter “a”. Student’s *T*-test was used to determine differences between two groups with significance levels as follows: **P* < 0.05, ***P* < 0.01, ****P* < 0.001. Data for Supplementary Figure S1, see Supplementary Table S6.
