# Supplementary figures and images for: Octopamine Shifts the Behavioral Response From Indecision to Approach or Aversion in Drosophila melanogaster
Source: Front Behav Neurosci. 2018 Jul 3;12:131. doi: 10.3389/fnbeh.2018.00131 (PMC6037846; doi:10.3389/fnbeh.2018.00131)

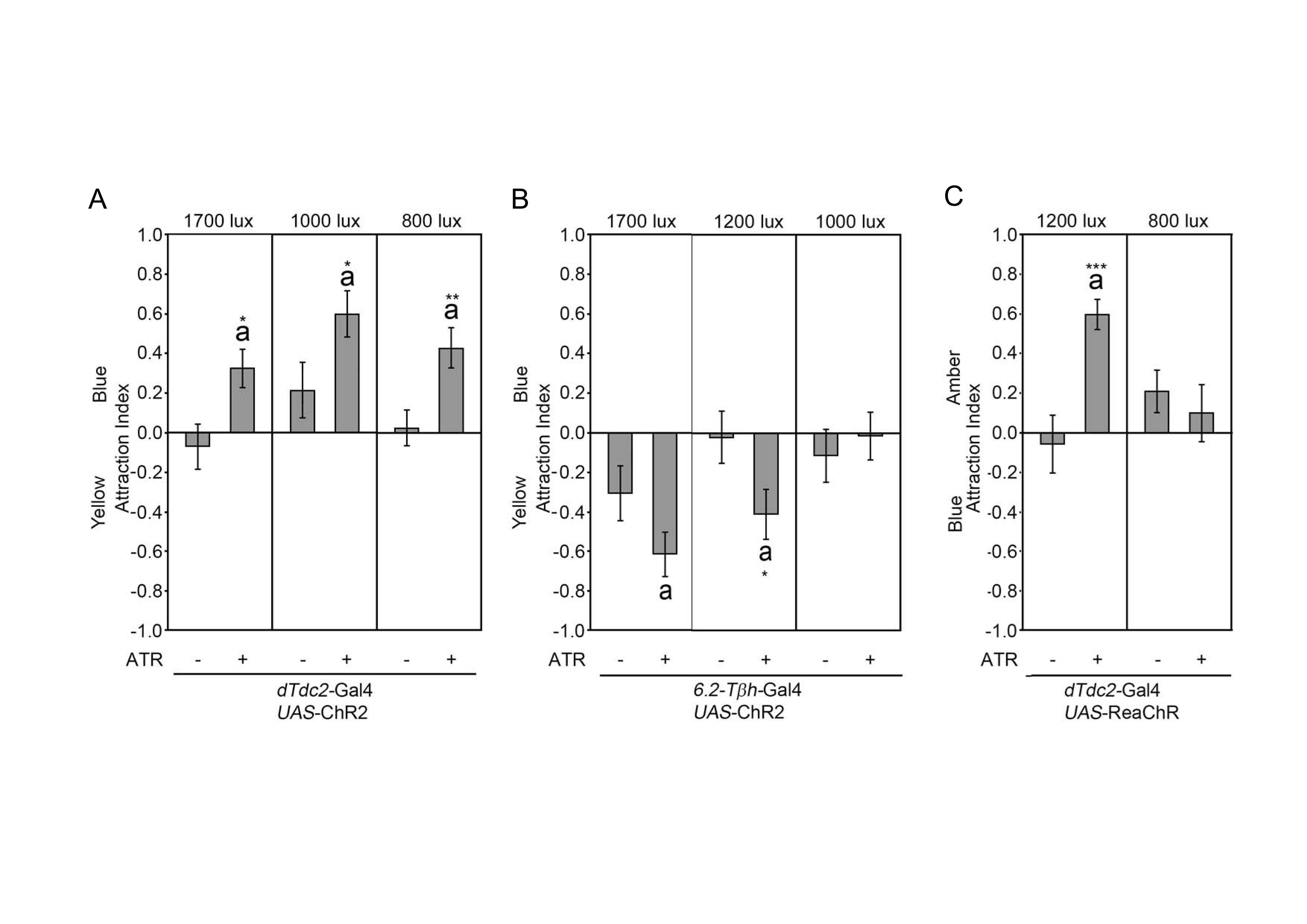

Supplement: FIGURE S1 — Effect of light intensity on behavioral outcome. The same flicker frequency of 2 s at 40 Hz, 16 s at 8 Hz was used for all experiments, but the intensity of both LEDs was changed simultaneously. (A) Different intensities indicated above the panel were used to activate neurons with a blue light-sensitive UAS-ChR2 under the control of the dTdc2-Gal4 driver. All the used intensities resulted in attraction for the site of activation (AIs for the 1700 lux control and experimental group: −0.07 ± 0.12 and 0.32 ± 0.1, n = 20, 20; for 1000 lux: 0.21 ± 0.14 and 0.6 ± 0.12, n = 34, 31; for 800 lux: 0.02 ± 0.09 and 0.43 ± 0.1, respectively, n = 39, 33). (B) Neurons activated with a blue light-sensitive UAS-ChR2 under the control of the 6.2-Tβh-Gal4 driver with different intensities showed significant aversion when illuminated with 1200 lux, but not 1700 lux or 1000 lux (AIs for the 1700 lux control and experimental group: −0.31 ± 0.14 and −0.62 ± 0.11, n = 20, 16; for 1200 lux: −0.02 ± 0.13 and −0.41 ± 0.13; n = 22, 19 and for 1000 lux: 0.12 ± 0.13 and −0.02 ± 0.12, respectively; n = 21, 19). (C) Activation with the amber-sensitive UAS-ReaChR in a dTdc2-Gal4-dependent manner leads to site attraction for the amber-illuminated site (AIs for the 1200 lux AIs of control and experimental group: −0.06 ± 0.15 and 0.6 ± 0.08, n = 18, 18; AIs for the 800 lux 0.21 ± 0.11 and 0.1 ± 0.14, respectively, n = 14, 5). Errors bars are SEM. The one-sample sign test was used to determine differences from random choice, and significant differences are labeled with the letter “a”. Student’s T-test was used to determine differences between two groups with significance levels as follows: *P < 0.05, **P < 0.01, ***P < 0.001. Data for Supplementary Figure S1, see Supplementary Table S6. [file Image_1.TIF]
